# Factors contributing to late breast cancer presentation for health care amongst women in Kumasi, Ghana

**DOI:** 10.4102/curationis.v38i1.1287

**Published:** 2015-08-31

**Authors:** Comfort Asoogo, Sinegugu E. Duma

**Affiliations:** 1 Division of Nursing and Midwifery, University of Cape Town, South Africa

## Abstract

**Background:**

Delay in presenting breast cancer for health care is dangerous because it can increase the mortality rate amongst affected women. Delaying health care and treatment makes it difficult to manage advanced breast cancer successfully. Understanding the factors that contribute to delays in presentation for health care can save lives.

**Objectives:**

The purpose of the study was to describe the factors which contribute to the late presentation of Ghanaian women with breast cancer for health care at a tertiary hospital in Kumasi, Ghana.

**Method:**

A descriptive qualitative research design was utilised to answer the research question: ‘What factors contribute to presenting with late breast cancer for health care amongst Ghanaian women who were treated for breast cancer at a tertiary hospital in Kumasi, Ghana?’ A sample of 30 women diagnosed with breast cancer and presented with Stage II and Stage III participated in the study. Semi-structured interviews and field notes were conducted for data collection. Content data analysis was used in line with the research question.

**Findings:**

Five themes were discovered as findings. These were: lack of knowledge about breast cancer; fear of cancer treatment and its outcomes; poverty; traditional and spiritual beliefs and treatments and caring for others.

**Conclusions:**

We recommend the development of breast cancer awareness programmes and health education at primary health care level.

## Introduction

Breast cancer is the most common cancer causing death in women, accounting for 16% of adult women's deaths worldwide (World Health Organization 2008). It is estimated that more than one million women worldwide are diagnosed with breast cancer annually (Ferlay *et al.* 2010). In the USA alone, breast cancer is the second leading cause of death from 10 common cancers in women after lung and bronchus cancer, and accounts for 15% of all deaths from cancer in women in that country (Jermal *et al.* 2009). A study on cancer mortality patterns reported breast cancer to be the most common cause of death in women at the Korle-Bu Teaching Hospital (KBTH) in Accra, Ghana (Clegg-Lamptey & Hodasi 2007). No literature was available on breast cancer screening practices amongst Ghanaian women.

According to the GLOBOCAN (2012), almost 50% of breast cancer patients, and 58% of breast cancer deaths, occur in the developing countries, thus making this a public health problem that needs urgent attention in these countries (Ferlay *et al.* 2010). Understanding factors that delay early diagnosis of this disease is thus vital in order to develop public awareness programmes to encourage women to seek health care early.

## Literature review

Breast cancer is an abnormal growth of the cells in the breast (American Cancer Society 2008). The TNM classification system is used to determine how far the cancer cells have spread to the nearby tissues. It evaluates the tumour size (T), involvement of regional lymph nodes (N), and distant spread of the disease (M) (Langhorne, Fulton & Otto 2007).

The absence of cancer registries in Africa and other developing countries has been reported to limit knowledge of the true incidence of breast cancer and burden of breast cancer in these countries (American Cancer Society 2008; Laryea *et al.* 2014). However, predictions of annual increases in the numbers of new breast cancer patients in African countries have been made available (Nggada *et al.* 2008).

Early diagnosis, increased access to mammographic screening, and access to effective drugs has been reported to improve mortality in developed countries such as the United States and Canada (Langhorne *et al.* 2007). The same cannot be said for developing countries where the breast cancer mortality remains high (WHO 2008) because of low screening and the absence of mammography for the early detection of breast cancer (Jermal *et al.* 2009). According to Harford (2011), although mammography is important in the early detection of breast cancer, it cannot detect all types of breast cancer. Therefore, public awareness about breast cancer, health education about self-breast examination, and seeking early health care remains the main strategies for the prevention of breast cancer.

Evidence shows that screening for breast cancer is influenced by the individual's socio-economic status. In Russia, women with high socio-economic status were reported to be seven times more likely to have mammograms than their poor counterparts. In Australia and Belgium, the women from low income communities were reported to be more likely to have mammograms than the wealthier women. Countries such as Portugal reported an overall low prevalence of screening (Jermal *et al.* 2009). The health professionals from both developed and under-developed countries need to understand what influences women to seek health care in their countries in order to develop specific prevention strategies for addressing breast cancer as a public health problem.

Literature highlights the importance of understanding the contributory factors to breast cancer for the development of prevention strategies of breast cancer as a public health problem. For instance, the Women's Health Initiative and Women's Intervention Nutrition studies in the USA helped in improving the understanding on the effects of dietary fat, obesity, and hormonal replacement therapy as contributory factors, and thus helped in increasing the effort of prevention and treatment of breast cancer to reduce the mortality in that country (National Cancer Institute 2005). Clegg-Lamptey and Hodasi (2007) reported that, despite various health educational programmes on breast cancer in Ghana, including the ‘Reach for recovery’ programme, Mammocare and health education on breast self-examination, at least 50% or more Ghanaian women with breast cancer still reported to hospital with an advanced stage of the disease. The same report highlights the need to investigate the factors that contribute to delay in seeking health care for breast cancer amongst Ghanaian women.

Increased risk of physical and psychological problems after diagnosis with breast cancer, generalised anxiety disorder, depression, difficulty in concentration, fatigue, negative thoughts, suicidal thoughts, uncertainty about treatment and fear about cancer recurrence and death have been reported amongst newly diagnosed breast cancer patients. These may also affect the entire family and could influence the delay in seeking health care for breast cancer (Al-Azri, Al-Awisi & Al-Moundhri 2009; Taleghani, Parsa & Nasarabadi 2006).

### Problem statement

One of the authors, a professional nurse in an oncology department at a tertiary hospital in Kumasi, observed that most of the women with breast cancer reported to the hospital with late stages of breast cancer. The causes for the delay in reporting for health care could not be easily determined from these patients or their relatives at the time of admission. Knowing that evidence from developed countries shows that, if detected early, breast cancer can be treated successfully (Langhorne *et al.* 2007), the authors undertook to investigate the factors that contribute to delay in seeking health care for breast cancer amongst Ghanaian women. Understanding the factors that contribute to delay in seeking health care amongst Ghanaian women was necessary to inform the development of strategies to promote women's early detection of breast cancer, and to encourage seeking health care on time.

## Purpose the study

The purpose of the study was to describe the factors which contribute to late presentation with breast cancer amongst Ghanaian women who were treated for breast cancer at a tertiary hospital in Kumasi, Ghana. The research question was ‘What factors contribute to presenting with late breast cancer amongst Ghanaian women treated for breast cancer at a tertiary hospital in Kumasi, Ghana?’

### Research setting

The study was conducted in a tertiary hospital, which is the second largest of the three tertiary hospitals in Ghana. The hospital is in Kumasi, the regional capital of the Ashanti Region in Ghana. The hospital has a 1000-bed capacity. Women make up 69.6% of cancer patients in Kumasi. Breast cancer, at 33.9% is the most common of all the cancers in the region, followed by cervical cancer at 29.4%, ovarian cancer at 11.3% and endometrial cancer at 4.5% (Kumasi Cancer Registry 2014). The latest development in the establishment of the cancer registry in Kumasi has made it possible to plan for services for the prevention and treatment of the common cancers in Kumasi (Laryea *et al.* 2012). The oncology unit of the hospital specialises in the provision of comprehensive cancer care for patients through haematology, medical and radiation oncology services. In 2008 (a year prior to the study), 8584 consultations were recorded at the outpatient outlet of the oncology unit (Komfo Anokye Teaching Hospital website).

## Research method and design

A descriptive qualitative research design was used because it is a holistic approach that is useful in exploring the behaviour, perspectives, feelings and experiences of people (Holloway & Wheeler 2002). The design was deemed appropriate to allow women with breast cancer to share their life stories and reasons for late reporting of breast cancer for health care.

### Study population and sampling

The population was all women with breast cancer, who presented to the tertiary hospital and were diagnosed with stage II and III of breast cancer. Convenience sampling was used to recruit women who were in the oncology department of the tertiary hospital and had presented, for the first time, at the clinic with breast cancer at stage II or later stages and had not been started on chemotherapy. This was the only inclusion criteria for the study.

The professional nurse in charge of the oncology department assisted the researcher to approach the patients for recruitment purposes. This mediated access ensured smooth recruitment of participants. A sample of 30 participants was recruited to obtain the wide range of views about the factors that contributed to delay in seeking health care for breast cancer.

### Pilot study

A pilot study was conducted to ensure that the interview questions were easily understood by participants and yielded appropriate data. Data from the pilot were included in the main data analysis because the participants shared similar characteristics with those in the main study. This is permitted in some qualitative research where there is no possibility for data contamination (Duma, Khanyile & Daniels 2009).

### Data collection process

Data collection was conducted between October and December 2009 using the semi-structured interview guide and probing questions in Twi, which is the common local language. The three main questions that guided the interviews were:

1. When did you first notice that there was something wrong with your breast?
2. What did you do to manage the abnormality in your breast?
3. Were there any factors which prevented you from seeking health care when you first discovered the abnormalities in your breast; if so, what were these factors?

The interviews were digitally recorded. Field notes were taken during the interview to capture relevant observations made during the interview session. The interviews were transcribed within 24 hours of the interviews.

The transcription was done in the participants’ language and later translated into English by the researcher, who is fluent in both Twi and English languages, and understood the common verbal and non-verbal communication in the community. A qualified and experienced Twi-English translator, who is from the same community as the participants, was appointed to check through both transcribed interviews for accuracy and possible loss of meaning. The translator identified minor English discrepancies between Twi and English direct translations. These were discussed with the researcher and corrected before data analysis was conducted.

Each transcribed interview was stored as a Microsoft Word document on the hard drive and backup flash drive according to the order and date in which the participants were interviewed.

### Data analysis

Content data analysis was conducted (Polit & Beck 2012). This included reading and re-reading each transcript to acquire a sense of the overall data and words, or statements that made sense in answering the research question. The transcripts were then read in totality to gain an overall impression of the data. Thereafter, transcripts were read again to highlight important statements that were related to the research question or seemed to be of interest. Different colour pens were used to mark the identified words or statements made by each participant. These different colours were used to assist the researcher to identify, organise, retrieve and analyse data. The colour codes were given meanings to ensure that the statements with similar meanings were clustered together. The statements were then grouped into clusters of meanings. Each cluster was coded as a theme cluster. Clusters with similar meanings were further analysed for meaning until the main themes emerged from the clusters. During the process of data analysis the researcher worked with the senior researcher (the corresponding author) to ensure credibility of the interpretation of themes and supportive raw data. Five themes emerged from the data. Later, the participants were contacted with the list of themes for member checking. These were explained in the Twi language as ‘groups of information’ that came from their participation in the research. Only 25 participants were reached for member checking. They all confirmed the themes as part of the information they had shared during the interviews.

## Findings

The sample was comprised of 30 women who had breast cancer and attended the oncology department for follow-up visits following initial diagnosis. Their ages ranged between 25 and 67 years, with the majority (60%) being 40 years old and above. The youngest participant, aged 25 years, was in her final year at the university and financially supported by her parents. Twenty-three participants were Christians and seven were of the Moslem faith. Only two participants were illiterate and had never been to school. Seven participants had primary level education, 16 obtained secondary and 5 women had obtained tertiary level education. Most of the participants were either unemployed or self-employed with only six participants who were unemployed and financially dependent on husbands or other family members. Eighteen participants sold vegetables at the market place to support themselves and their families. Of those who reported to be employed, two were employed as school teachers, one was employed as a secretary in a local industry, one was employed as a shop assistant and one was employed as a waitress at a restaurant. This data reflects the social and economic situation of people in Kumasi Metropolis.

The main findings of the study are described as the five themes that emerged from content data analysis. The extracts and quotes are used to support the development of the themes.

### Lack of knowledge about breast cancer

The theme, lack of knowledge about breast cancer, was attributed to the recognition of abnormalities in the breast as normal female developmental stages such as menopause, breast feeding and having not breast fed; painless symptoms and having never seen anyone with such symptoms in the community before.

One participant associated the early symptoms of breast cancer with breast-feeding:

‘I thought it was normal. All women always have it like that and then when the baby sucks, then it goes away. So I thought it would go away too.’ (P1)

Another participant highlighted this as follows:

‘I thought it was menopause because my friend told me that you can experience many things when that time comes. I really thought that I have reached that age.’ (P3)

Another participant highlighted lack of knowledge about the symptoms of breast cancer as follows:

‘I first felt a lump in my breast, very hard like a stone and big but it was not painful so I ignored it. I never took it seriously at the time. It became very heavy, but there was still no pain as such, just hardness and heaviness. Then later I came to the breast clinic.’ (P7)

The following extract from one participant highlights lack of knowledge about breast cancer symptoms:

‘This type of disease is very dangerous it does not cause pain. For some time I could not even feel the lump again so I thought it had gone. Meanwhile it was there, eating me up. For a long time there was no pain. Then it started all of a sudden again and this time I knew that I had to go to the doctor.’ (P2)

Having never seen anyone with breast cancer was also identified as lack of knowledge about breast cancer, as indicated in the following extracts of data:

‘I have never heard of that disease in all my life, no one has this in my village or family … I wish someone told me about it.’ (P21)

‘Hey, I have never seen someone with this disease before in my home. How would I have known that it was a bad disease, cancer? I just thought it would go away.’ (P27)

A young participant demonstrated her lack of knowledge about breast cancer as follows:

‘Hmm! I still cannot believe that it is breast cancer, it is only God who knows better. At my age [*25 years old*], how could it be … but then that is what I was told here, so now I must accept it as cancer.’ (P11)

### Fear of cancer treatment and its outcomes

The expressions of fear of surgical procedures were found in the participants’ use of words or phrases, such as ‘chopping my breast off’, ‘cutting me up’, and ‘amputating me’. These words were interpreted as real fear of treatment and its outcome and were considered to be real contributions to the delay in seeking health care.

The following extract from data shows this fear:

‘I was told that if I take it to the hospital the doctor will definitely chop it off and let me die. So I thought I could delay that a bit … who wants to die?’ (P18)

Another statement demonstrating fear of surgery was the following:

‘I could not let myself come for that. I know someone who died after she was cut open when she gave birth. They say she bled until there was no blood inside her. I could not do that to myself … I stayed for another year or so with this thing oozing.’ (P23)

The fear of the effects of chemotherapy was also demonstrated in the data as follows:

‘I was told that cancer medicine kills everything – good and bad inside you. I decided to keep living and not kill myself with those dangerous medicines.’ (P1)

Fear of the outcome of breast cancer treatment as a reason to delay reporting was also expressed in the following extract:

‘I was so worried about what will happen to me if the doctors found this to be cancer. I do not even have a child yet. What if they want to remove all my breasts, will I ever be a woman again? So I just kept this thing to myself and prayed to fall pregnant first so I could breastfeed my baby.’ (P9)

### Poverty

Lack of money was an issue, especially for those coming from afar and who were referred to the tertiary hospital, as demonstrated in the quotes below:

‘Last year, the doctor at the district asked me to come to Gee, but I did not have enough money to come then I was also told that the national health insurance does not cover all the medicines for this.’ (P15)

‘I did not have enough money to come until now. I tried to save some money from last harvesting and selling stuff so that I could come only now.’ (P11)

The following extract also showed the lack of money as a reason for delaying reporting for health care:

‘I started saving some money from sales at the market place so I could come to the hospital. You know it is hard for everybody these days, so you cannot ask for help anywhere.’ (P18)

The following expression also described a participants’ lack of money or poverty:

‘I did not have money last summer. My husband is very old to work. The little money that we had was all used for herbal medicine because they do not cost very much.’ (P27)

### Traditional and spiritual beliefs and treatments

Traditional and spiritual beliefs and their related treatments featured prominently in data as factors that contributed to the delay in presenting for health care. These were clustered as any form of belief and treatment that differed from Western medical interventions for breast cancer as demonstrated in the following extracts:

‘My mother also had breast cancer and the doctor cut off both her breasts. She died some years ago. We did not want the same thing to happen to me. So we thought this time it could be helped with the traditional ways. We contacted a traditional healer and prayed to the ancestors for traditional medicines to work.’ (P19).

‘I thought the herbs from the traditional healer could help me. Later when I noticed there was no improvement and I had severe and continuous pain in my breast I decided enough was enough and came here.’ (P26)

One other extract demonstrated spiritual beliefs as a contribution to delaying seeking health care:

‘I went to the prayer camp for almost the whole year but I did not see any changes. I went around prayer camps, hoping for spiritual healing. Other people get healed from these prayers camps. I thought I could too. Finally I lost faith and reported at the District hospital and they referred me here.’ (P13)

Another participant responded on traditional medicines as follows:

‘Last year, my son took me to the traditional doctor, but he did not help. He then brought me to hospital at the district and the doctor said they would have to cut off my breast but I refused. We returned home to continue with the herbal medicines from the traditional doctor. My son left for work [*he works in Accra*]. When he came home again and saw that there was no improvement, he brought me here as the sore was not healing.’ (P1)

### Caring for others

The participants had to look after the needs of others whilst neglecting their own health needs as demonstrated by the extracts below:

‘I could not leave the children alone and come this far. At least now my daughter has someone to stay with them and she brought me here.’ (P14)

Another participant expressed this as follows:

‘I had to take care of my husband. He has stroke. My daughter-in-law helps, but she also has her own children. Because this was not very painful, I had to look after him. I see that I should have come earlier.’ (P19)

‘If you are a mother and your children need you, it's not easy to pack and leave them. My son was writing exams. I needed to make sure that he was well taken care of so that even if I die, he would have passed his school exams. Then he can look after himself.’ (P6)

The findings demonstrate the different factors that contributed to the delay amongst women in reporting for health care. Understanding these factors is useful for the development of public awareness and health education programmes, including self-examination of the breast and reporting any abnormalities discovered on the breasts for immediate health care.

## Ethical considerations

The study met all of the Helsinki Declaration requirements (World Medical Association 2013). The ethical approval was received from the university ethics committee (REC REF. 467/2009). The permission to conduct the study was also received from KATH/Ministry of Health and the head of department of the oncology directorate.

Informed consent for voluntary participation and withdrawal from the study and permission to use a digital recorder to record the interviews was obtained from the participants. Data management procedures were applied to safeguard the privacy and confidentiality of the participants’ data as proposed by Leedy and Ormrod (2005). Debriefing sessions for participants who showed emotional distress were arranged with the local hospital psychologist, but no participant needed referral to the psychologist.

### Trustworthiness

Trustworthiness was ensured by verification through member checking, which was conducted with 25 of the 30 participants who could be reached in March 2010. Member checking means returning to the research participants with the findings or themes to verify whether the findings match with the description of their views (Polit & Beck 2012). All participants who were contacted confirmed that the themes presented to them by the researcher were a reflection of their views. An audit trail was kept for anyone wanting to verify the findings or wanting to repeat the study in another setting.

## Discussions

The findings revealed that considerable time for the participants had elapsed between identifying abnormalities in the breast and reporting to the hospital. The authors identified five main themes or factors that contributed to the delay in seeking health care for breast cancer. Delays in reporting for health care were reported in other developing countries where at least 75% of women with breast cancer were, for the first time, diagnosed in the clinical stages III and IV of the disease (Clegg-Lamptey & Hodasi 2007; Tjemsland & Soreide 2004).

The lack of knowledge about breast cancer as a factor contributing to late presentation for health care is of great concern because it indicates that the breast cancer awareness campaigns are either not working or are not reaching the target population, such as women in the rural areas. Clegg-Lamptey and Hodasi (2007) found that about 50% or more Ghanaians with breast cancer still reported to the hospital with an advanced stage of the disease. This situation occurred despite various health education programmes on breast cancer in Ghana, including the ‘Reach for Recovery’ programme, Mammocare and health education on breast self-examination. Tjemsland and Soreide (2004) also reported that, despite all the efforts to combat breast cancer, the recent public concern about the disease and its destructive consequences on women in their reproductive years is rising. The level of awareness about the disease remains low with low survival rates in some communities.

The findings reveal that only the surgical treatment of breast cancer is known and feared by the Ghanaian women. This fear is a major factor that contributes to the delay in reporting for health care for breast cancer. The health professionals have a responsibility to teach the public about the different types of treatment for breast cancer, including the use of chemotherapy, which is an effective treatment for breast cancer (Kuo *et al.* 2008). Similar findings regarding the fear of death as a result of surgical treatment of breast cancer have been acknowledged in literature (De Groot *et al.* 2005). Such fears are as a result of hearsay and need to be corrected. The public should be informed that deaths related to breast cancer are attributed to the advanced stages of the disease and reporting that is too late for medical care, when little can be done to save the patients’ lives.

Poverty and financial difficulties, such as unemployment, has been reported as an issue for the delay in seeking general health care in both developed and developing countries (Anyanwu 2008; Arndt *et al.* 2002; Montazeri *et al.* 2003). Poverty is therefore a major factor that contributes to the delay in seeking health care for breast cancer in Ghana, because the country remains largely poor, although there have been some improvements since the 1990s (Coulombe & Wodon 2007). Other indicators of poverty, such as the long distance travelled by participants to the referral hospital and related transport fees, are other factors that contribute to the delay in reporting for health care for breast cancer. Similar conclusions have been made by Anyanwu (2008) as common factors that contribute to late presentation for health care in the developing countries. Castro-Leal *et al.* (2001) also found that some poor households face long distances and high costs to access health care centres and that these may lead to delays in seeking health care. Similar conclusions could be made from the findings of this study.

Caring for others, such as an ill spouse or children and grandchildren, were also identified by Ghanaian women as factors that contribute to delay in seeking health care. Other studies (Arndt *et al.* 2002; Burgess *et al.* 2006) have shown that women living in large households were reported to be at high risk when presenting with a late stage of breast cancer. Women who cared for large families with children were particularly at risk. Awareness campaigns to inform women that neglecting their personal health whilst caring for others is dangerous for both themselves and their loved ones. There is a risk that they could die and not be able to care for their families.

Traditional and spiritual beliefs and related treatments featured strongly in the findings as the factors that contributed to the delay in seeking health care for breast cancer in Ghana. Traditional and spiritual beliefs and related treatments are entrenched in the Ghanaian culture, where at least 70% of the population is reported to be exclusively dependent on traditional medicine for their health care (World Health Organization 2001). The *Ghana Traditional Medicine Act*, 575 of 2000 regulates the sale, use and practice of traditional medicine by registered traditional medicine practitioners. The country's earlier declarations, including the Dental Decree and the Nurses and Midwives Decree of 1972 both allowed the use and practice of traditional medicines in Ghana, provided that there were no life-endangering procedures used (WHO 2001). Similar findings have been reported in the African countries (Al-Azri, Al-Awisi, Al-Moundhri 2009; Anyanwu 2008). We argue that the use of traditional medicines per se is not a bad idea, but traditional healers should be encouraged to work closely with other health professionals and refer the patients when there is no improvement noted within a reasonable time. The patients also have a responsibility to ensure that they do not waste time following different spiritual healers or traditional healers before considering reporting to the hospital for health care.

## Limitations

The qualitative nature of the study and the fact that the study was conducted in one hospital setting are limitations in that the findings are not generalisable. The use of these findings for generalisation purposes should be handled with caution as this was not a community-based study.

## Recommendations

Public awareness programmes about breast cancer, including early detection through personal breast examination, seeking health care early and dispelling all myths about breast changes as normal physical development should reach all communities, including rural communities. Capacity development on early detection, timely management or referral procedures should be provided to all health professionals at primary health care institutions. Research on women who present early for health care should be conducted to develop best practice models for early detection of breast cancer.

## Conclusion

The causes for delay in reporting for health care for breast cancer are varied, but they all hinge on the women's lack knowledge about breast cancer and not recognising the seriousness of the disease. The five themes are by no means conclusive of all the factors that contribute to the late presentation for breast cancer management amongst Ghanaian women. Nonetheless, they provide a good starting point for health professionals to identify areas to focus the awareness campaigns and health education programmes.
